# A Multi-Parametric Investigation into Collector Adsorption and Its Role in the Chromite-Olivine Flotation System

**DOI:** 10.1021/acsomega.5c09104

**Published:** 2025-12-04

**Authors:** Savas Ozun, Rahman Raimov

**Affiliations:** 52994 Süleyman Demirel University, Faculty of Engineering and Natural Sciences, Department of Mining Engineering, Isparta 32260, Turkiye

## Abstract

Chromite ores that are difficult to beneficiate by gravity methods often necessitate flotation, particularly when fine particle liberation or overgrinding complicates separation. This study presents a comparative assessment of the flotation behavior of chromite and olivine through systematic microflotation tests, complemented by thermogravimetric analyses (TGA) and Fourier Transform Infrared (FTIR) spectroscopy to elucidate collector–mineral interactions. The effects of collector type (amine and sulfonate), pulp pH, collector concentration, air flow rate (AFR), conditioning time, and particle size were comprehensively investigated. Results indicated that both minerals responded similarly to amine, achieving maximum recoveries of about 80% for chromite and over 90% for olivine within the pH range of 7–8. Sulfonate proved most effective under strongly acidic conditions (pH < 2), with recoveries exceeding 75%. Across all conditions, the majority of flotation occurred within the first 30 s. Flotation efficiency improved with increasing AFR but declined with coarser particle sizes, while conditioning time had a negligible impact. TGA of the collector solutions, prepared and conditioned with each mineral at their respective optimum pH values, revealed distinct differences in thermal decomposition behavior compared with the unconditioned solutions and distilled water. These variations indicate that interactions occurred between the collector molecules and the mineral surfaces. FTIR spectra of collector-conditioned minerals rinsed with distilled water further revealed the absence of characteristic C–H peaks, indicating that adsorption was predominantly physical rather than chemical in nature.

## Graphical abstract

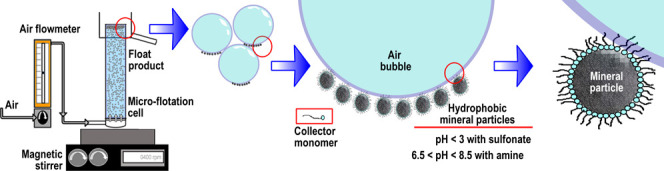

## 1 Introduction

The beneficiation of chromite ores represents a critical industrial process, given that only ores with a minimum Cr_2_O_3_ content of approximately 32% qualify for direct metallurgical use. Lower-grade ores require enrichment to meet these specifications. Currently, gravity separation dominates industrial practice due to the substantial density difference between chromite and gangue minerals, while magnetic separation is utilized as a secondary option given chromite’s weak paramagnetic property.^1,2^ However, these conventional methods exhibit diminished efficiency when processing finely liberated ores, often leading to the loss of valuable fines as tailings.^3,4^

In this context, flotation has emerged as a promising alternative or complementary technique for selectively separating chromite from silicate gangue minerals such as olivine and serpentine. Research efforts have focused on identifying optimal flotation parameters and reagent regimes. Initial investigations by Sobieraj and Laskowski (1973)^5^ revealed the pH-dependent recovery behavior of Russian chromite with various collectors, while Doğan (1975)^6^ achieved selective chromite suppression using sodium tartrate at pH 5–7, and Gallios et al. (2007)^7^ examined the relationship between pH, collector concentration, and depressant effectiveness in chromite-serpentine separation. Additional contributions by Ağaçayak et al. (2006),^8^ Seifelnassr and Tamam (2011),^1^ and Turri et al. (2017)^9^ highlighted the significance of reagent selection, pulp density, and conditioning parameters, achieving recoveries of 65–90% under optimized conditions. Recent pilot and industrial-scale studies have demonstrated the potential of flotation for recovering chromite from waste streams, although selectivity challenges persist.^4,10^ Despite extensive research, significant knowledge gaps remain in chromite-olivine selective flotation. Comparative evaluations of multiple collectors under systematically varied conditions are limited, and the fundamental adsorption mechanisms governing collector–mineral interactions remain insufficiently characterized. Few studies have employed spectroscopic techniques such as FTIR to correlate surface chemistry with flotation outcomes.^11^ Consequently, the fundamental basis for selective separation between chromite and olivine—minerals with similar surface properties—remains inadequately understood.

This study addresses these knowledge gaps through a comprehensive experimental program designed to optimize chromite–olivine flotation comparatively. Using microflotation techniques, we systematically investigated the effects of collector type and concentration, pulp pH, AFR, conditioning time, and particle size distribution for both minerals. Additionally, FTIR spectroscopic analysis provided insights into molecular–level interactions between collectors and the respective mineral surfaces. By integrating flotation performance data with adsorption characterization, this research aimed to develop a mechanistic understanding of collector selectivity and to identify optimal operational parameters for the efficient separation of chromite and olivine. Beyond laboratory-scale optimization, the findings also have valuable implications for processing plants, where inefficient beneficiation and overgrinding frequently generate fine-sized tailings that are sent to storage despite exhibiting grades comparable to those of run-of-mine ore. Thus, by addressing chromite and olivine in a comparative framework, this study not only advances the scientific understanding of flotation mechanisms but also highlights practical pathways for recovering these minerals from industrial tailings, contributing to improved resource efficiency and sustainable mineral processing.

## 2 Materials and Methods

### 2.1 Materials

#### 2.1.1 Mineral Samples

Chromite and olivine concentrate samples were obtained from Elazığ, Türkiye. XRD analyses were conducted to determine the mineralogical composition of the as-received samples. As shown in Figure 1, the XRD pattern of the chromite sample indicates chromite as the dominant mineral phase, with forsterite (olivine) and serpentine (chrysotile) present as minor phases. Similarly, the XRD results for the olivine sample (Figure 2) reveal that it is mainly composed of olivine (forsterite), accompanied by small amounts of serpentine (chrysotile). The samples were subsequently subjected to sieve analysis to obtain three particle size fractions: −300 + 212 μm, −212 + 150 μm, and −150 + 106 μm. The elemental composition of each size fraction was then determined by XRF analysis, and the results are presented in Table 1.

**Figure 1:**
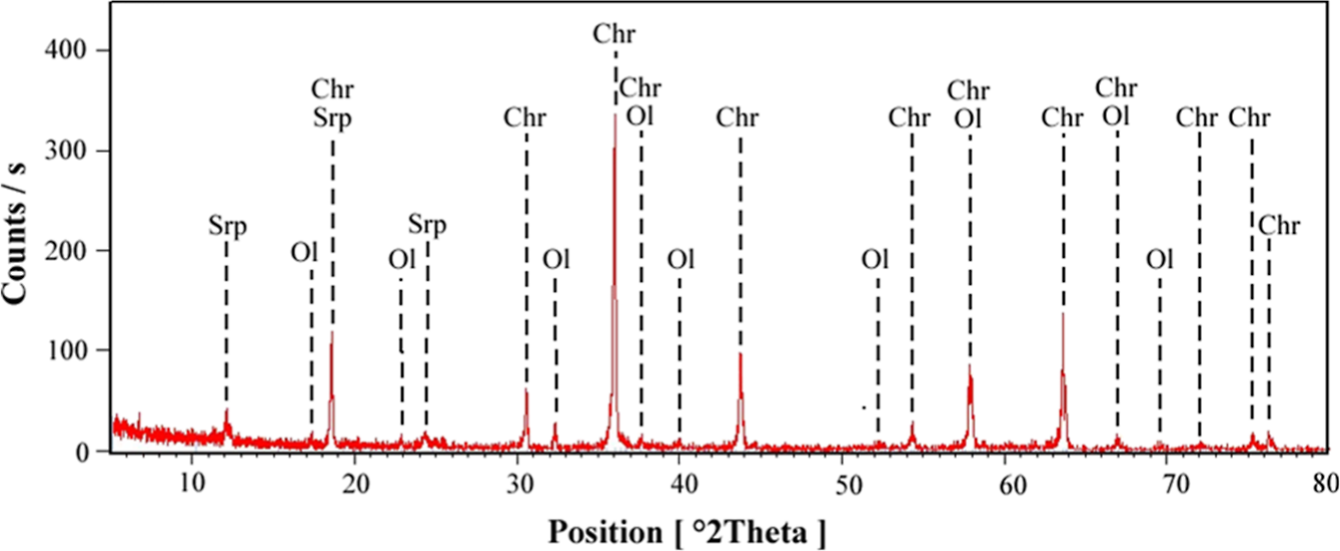
X-ray pattern of chromite sample (Chr: chrome spinel, Ol: olivine, Srp: serpentine).

**Figure 2:**
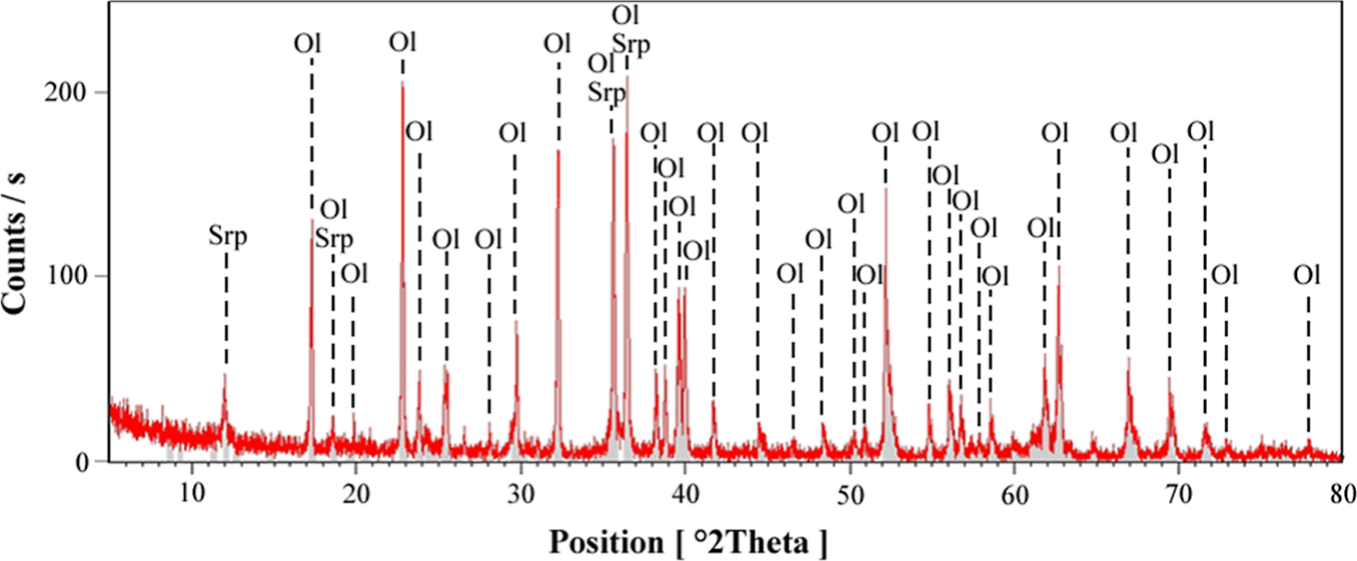
X-ray pattern of olivine sample (Ol: olivine, Srp: serpentine).

**Table 1:**
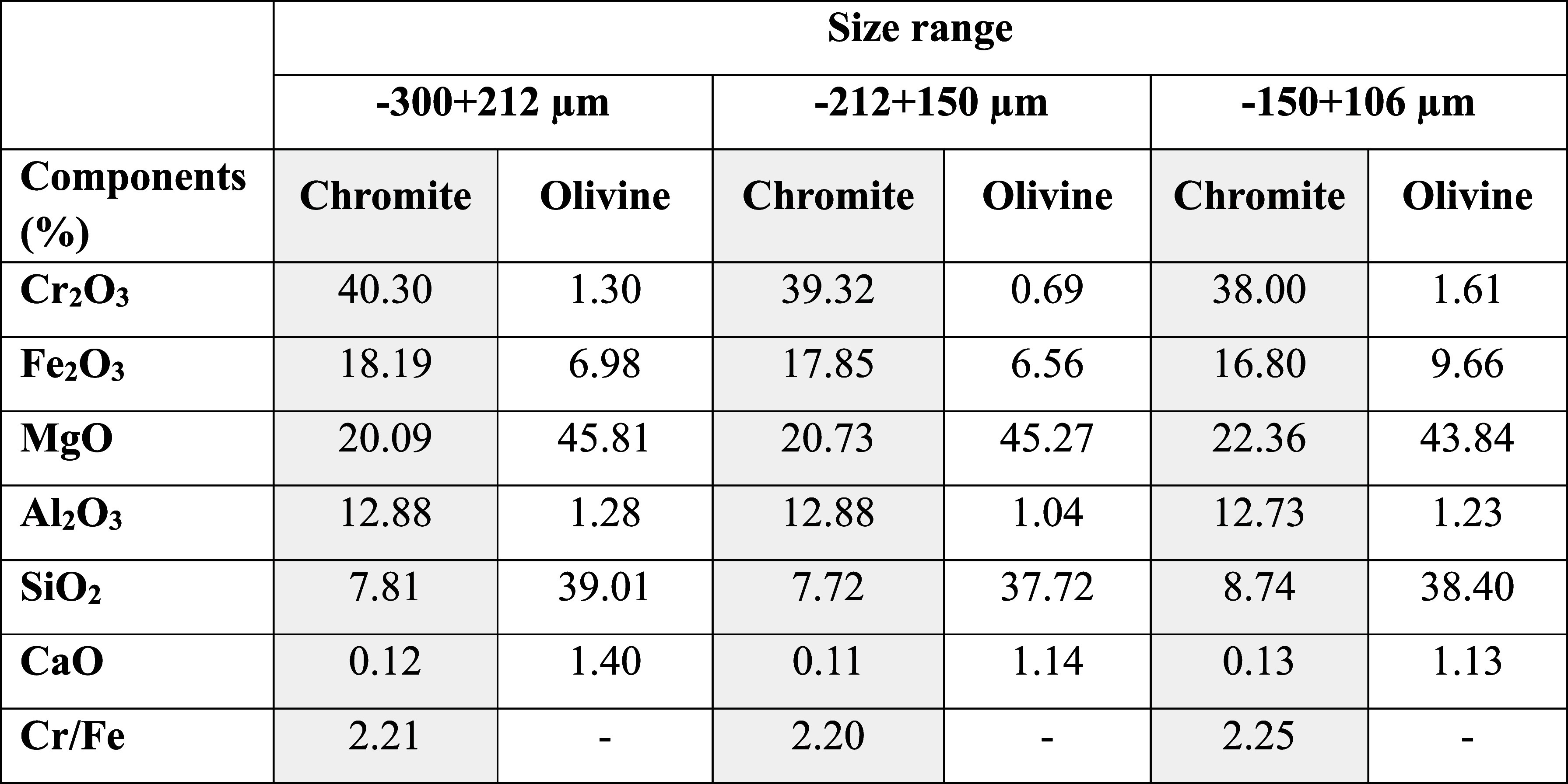
Chemical Analysis of Chromite and Olivine Samples

#### 2.1.2 Reagents

The collectors utilized in this study were a cationic amine (Aero 3000C) and an anionic sulfonate (Aero 825), all procured from Cytec Solvay Group. Stock solutions of each collector were prepared by dissolving the reagent in distilled water to achieve concentrations of 2 g/L. Before each microflotation test, these stock solutions were freshly diluted with distilled water to obtain working concentrations in the range of 7.5–20 mg/L. The solutions were homogenized using a magnetic stirrer to ensure uniformity. The pH of the aqueous medium was adjusted using analytical grade hydrochloric acid (HCl) and sodium hydroxide (NaOH), both supplied by Merck, to create acidic and basic conditions, respectively.

### 2.2 Methods

#### 2.2.1 XRD Analysis

The mineralogical composition of the as-received chromite and olivine concentrate samples was determined using X-ray diffraction (XRD) analyses. Measurements were performed on a Panalytical PW3040/60 X’Pert Pro MPD diffractometer, with Cu Kα radiation (λ = 1.5406 Å) under standard operating conditions. The obtained diffraction patterns were used to identify the crystalline phases present in the samples.

#### 2.2.2 Zeta Potential Measurements

The zeta potential (ζ) of chromite and olivine samples was measured across a range of pH values to determine their isoelectric points (pH_IEP_; IEP). Measurements were carried out using a Malvern Zetasizer Nano ZSP, which determines zeta potential by measuring the electrophoretic mobility of mineral particles (3.8–100 nm) under an applied electric field. For each measurement, a pulp was prepared by dispersing 10 mg of a representative mineral sample in 100 mL of distilled water at ambient temperature. After conditioning the suspension for 5 min at the target pH, it was transferred to the sample cell for analysis. The reported zeta potential for each pH value represents the mean of at least three replicate measurements obtained from the same sample. The standard deviations were typically within ± 1–2 mV, consistent with the expected reproducibility of the instrument. The low variability among replicates indicates high measurement precision and confirms the reliability of the reported zeta potential data across the investigated pH range.

#### 2.2.3 Microflotation Tests

Microflotation tests were conducted to evaluate the flotation response of chromite and olivine samples as a function of froth collection time and key operating variables, including pH, collector type and concentration, conditioning time, AFR, and feed particle size range. Mineral samples within the particle size fraction of −212 + 150 μm were used in all microflotation experiments, which were conducted both in the presence and absence of a collector. The experimental setup employed in this study is schematically illustrated in Figure 3.

**Figure 3:**
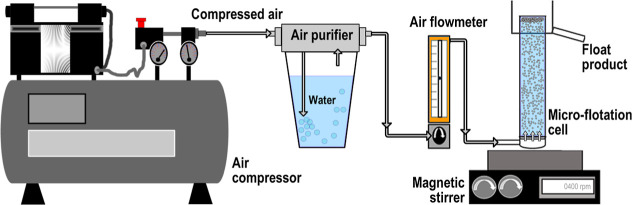
Microflotation test setup.

The experimental procedure for each microflotation test involved conditioning a 1 g mineral sample in 80 mL of an aqueous solution containing the collector at a specified concentration and pH. After conditioning, the pulp was transferred to the flotation cell, and flotation was initiated by introducing a steady stream of air. The froth products were collected at 15, 30, 60, and 120 s from the start of aeration. The collected concentrates were then filtered, dried for 24 h at 100 °C ± 5 °C, and weighed to determine their mass. Each microflotation test was repeated three times under identical conditions, and the reported results represent the average values of these replicates. The flotation recovery (R %) was calculated based on the dry mass of the products using eq 1

1 $$R\left(\%\right)=\left(\frac{C}{F}\right)\times 100$$

where *C* is the mass of the dried concentrate and *F* is the initial mass of the feed sample (1 g). The cumulative recovery at the given time was determined by summing the recoveries of all products collected up to that point.

#### 2.2.4 TGA Analysis

TGA analyses were performed using a Polymer Laboratories PL-1500 thermogravimetric analyzer. Approximately 10 mg of each sample, obtained from the respective collector solutions after the conditioning of chromite and olivine at their optimal pH values, was placed in a platinum crucible. The samples were heated from room temperature to 170 °C under an air atmosphere at a constant heating rate of 15 °C min^–1^. The mass loss as a function of temperature was recorded to evaluate the thermal stability and decomposition behavior of the conditioned samples.

#### 2.2.5 FTIR Analysis

The FTIR spectroscopy was utilized to analyze the surface of both the original mineral samples and the minerals after separate conditioning with each collector. The spectra were acquired in the mid-infrared region (4000–400 cm^–1^) using a Jasco V-770 UV–vis–NIR spectrometer equipped with an Attenuated Total Reflectance (ATR) accessory. All measurements were performed at ambient temperature, with each final spectrum representing an average of 200 scans recorded at a resolution of 4 cm^–1^.

## 3 Results and Discussion

The selection of collectors for the subsequent flotation tests was based on well-established principles of mineral surface chemistry. The floatability of minerals is primarily controlled by their pH-dependent surface charge. Below its IEP, a mineral surface is positively charged, facilitating the electrostatic adsorption of anionic collectors. Conversely, at pH values above the IEP, the surface becomes negatively charged, creating favorable conditions for the adsorption of cationic collectors.^12^ Based on this theoretical framework, the subsequent experimental work systematically evaluated the effectiveness of a cationic amine and an anionic sulfonate to explore these distinct adsorption pathways.

Initial microflotation tests were performed in the absence of collectors to establish the natural floatability of chromite and olivine as a function of pH. Across the entire pH range investigated, both minerals exhibited negligible recovery, confirming their inherently hydrophilic nature and the necessity of a collector to induce flotation.

### 3.1 Zeta Potential Measurements

The zeta potential results presented in Figure 4a,b show that both chromite and olivine exhibit a similar trend in distilled water, with the surface charge shifting from negative to positive as the pH decreases. Their IEP values were determined to be approximately pH 6, where the zeta potentials approach zero. At higher pH values (>6), the minerals exhibit distinctly negative surface charges (approximately −29 mV for chromite and −20 mV for olivine), while at lower pH values (<6), their surfaces become progressively positive, reaching up to +30 mV for chromite and +20 mV for olivine. These findings indicate that chromite and olivine display comparable electrokinetic behavior, with IEPs occurring within the same pH region. The experimental results are consistent with previously reported literature data.^13–16^

**Figure 4:**
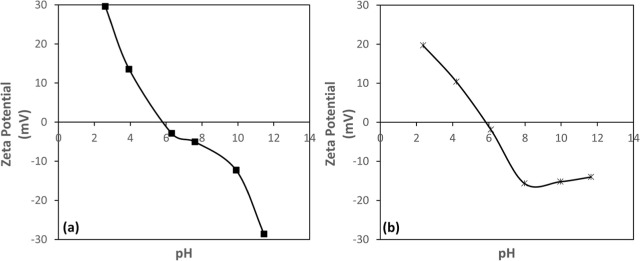
pH-dependent zeta potential of (a) chromite and (b) olivine.

### 3.2 Effect of Collector Type-pH Change on Flotation Efficiency

This section presents a detailed investigation of the pH-dependent flotation behavior of chromite and olivine in the presence of two distinct types of collectors: an anionic and a cationic reagent. The experimental conditions and parameters employed in the microflotation tests are summarized in Table 2.

**Table 2:** Variables Applied in Collector vs pH-Dependent Flotation Tests

**flotation variables**	**values**
mixing rate	1200 rpm (conditioning) 400 rpm (flotation)
conditioning time	5 min
AFR	1.6 L/h
flotation periods	15, 30, 60, 120 s
collector concentration	amine 7.5–20 mg/L sulfonate 10–20 mg/L

#### 3.2.1 Effect of Amine Type Collector-pH Change on Flotation Efficiency

The effect of amine-type cationic collector on the flotation recovery of chromite and olivine was investigated as a function of pH. The distribution and speciation of amine collectors within aqueous media demonstrate pronounced pH dependency. Under acidic conditions, the protonated cationic species (RNH_3_^+^) predominate, with their activity gradually declining as pH rises above neutrality. In strongly alkaline environments, the neutral molecular form (RNH_2_) becomes prevalent, while the pH region is characterized by the potential formation of ion–molecular complexes (RNH_2*_RNH_3_^+^). This speciation profile reflects a dynamic equilibrium that responds systematically to variations in solution chemistry, particularly hydrogen ion concentration.^12^

As shown in Figure 5a,b, flotation recovery was minimal at pH values below 6 for both minerals. In this acidic range, the mineral surfaces were positively charged (Figure 4a,b)—similar to the collector—leading to electrostatic repulsion. However, as the pH increased, flotation recovery rose significantly, reaching an optimum in the pH range of 7–8. This behavior is consistent with the surface charge characteristics of the minerals, which became progressively more negative above their IEP at approximately pH 6. The enhanced recovery observed within the optimal pH range can be attributed to the strong electrostatic attraction between the negatively charged mineral surfaces and the protonated amine species. Specifically, at pH 7–8, the chromite surface acquires a predominantly negative charge due to hydroxyl ion adsorption, which in turn promotes the electrostatic attachment and adsorption of the positively charged amine collectors. Conversely, at higher pH values, the chromite surface becomes progressively covered by metal hydroxide species such as Cr­(OH)_3_ and Fe­(OH)_3_, which can partially neutralize the surface charge and inhibit the adsorption of amine monomers. The marked decline in flotation efficiency beyond the optimum pH range is therefore attributed to both the surface change effect and the reduced concentration of protonated amine cations (RNH_3_^+^)—the active collector species—at elevated pH levels.^12,17^

**Figure 5:**
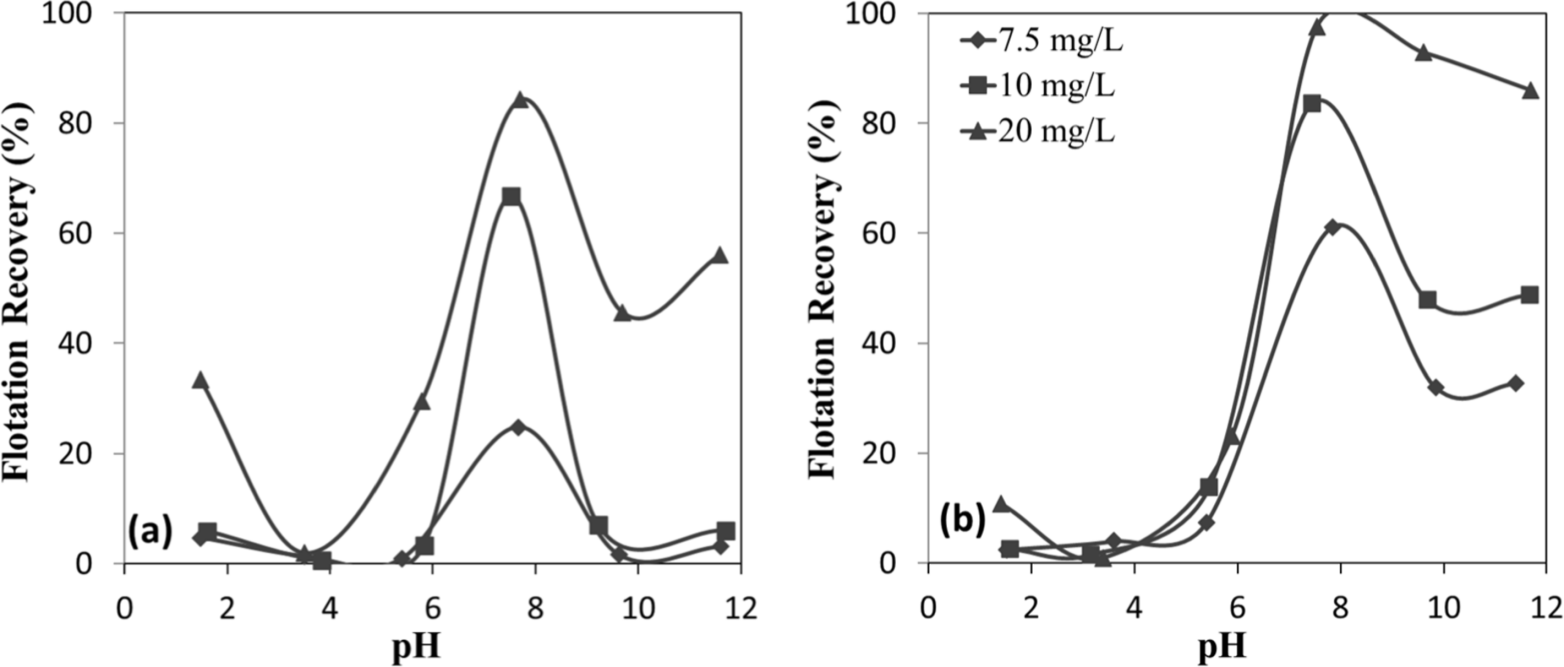
pH-dependent flotation recovery of (a) chromite and (b) olivine with amine.

The flotation responses of the two minerals showed marked differences as a function of collector concentration and froth collection time. Across all experimental conditions, olivine (Figure 5a) consistently achieved higher flotation recoveries than chromite (Figure 5b), with maximum performance for both minerals determined near pH 8. Although olivine displayed less negative zeta potential values compared to those of chromite (Figure 4a,b), its enhanced floatability can be attributed to its surface chemistry, which promoted stronger electrostatic interactions with amine collectors compared with chromite. Kinetic tests conducted under optimal conditions (pH 7–8, 7.5–20 mg/L collector concentration) further confirmed these differences. At the lowest collector concentration (7.5 mg/L), the contrast was pronounced, with recoveries after 30 s reaching only ∼17% for chromite (Figure 6a), compared to ∼54% for olivine (Figure 6b). Although the gap narrowed at higher collector concentrations, it remained significant; at 20 mg/L and 120 s, recoveries were about 85% for chromite and over 97% for olivine. While chromite recoveries increased gradually and plateaued at lower values, olivine recoveries rose sharply and approached near-complete flotation within a short time (as early as 15 s). These findings demonstrate the stronger affinity of the amine collector for olivine surfaces and highlight the potential for selective separation of olivine from chromite in flotation processes.

**Figure 6:**
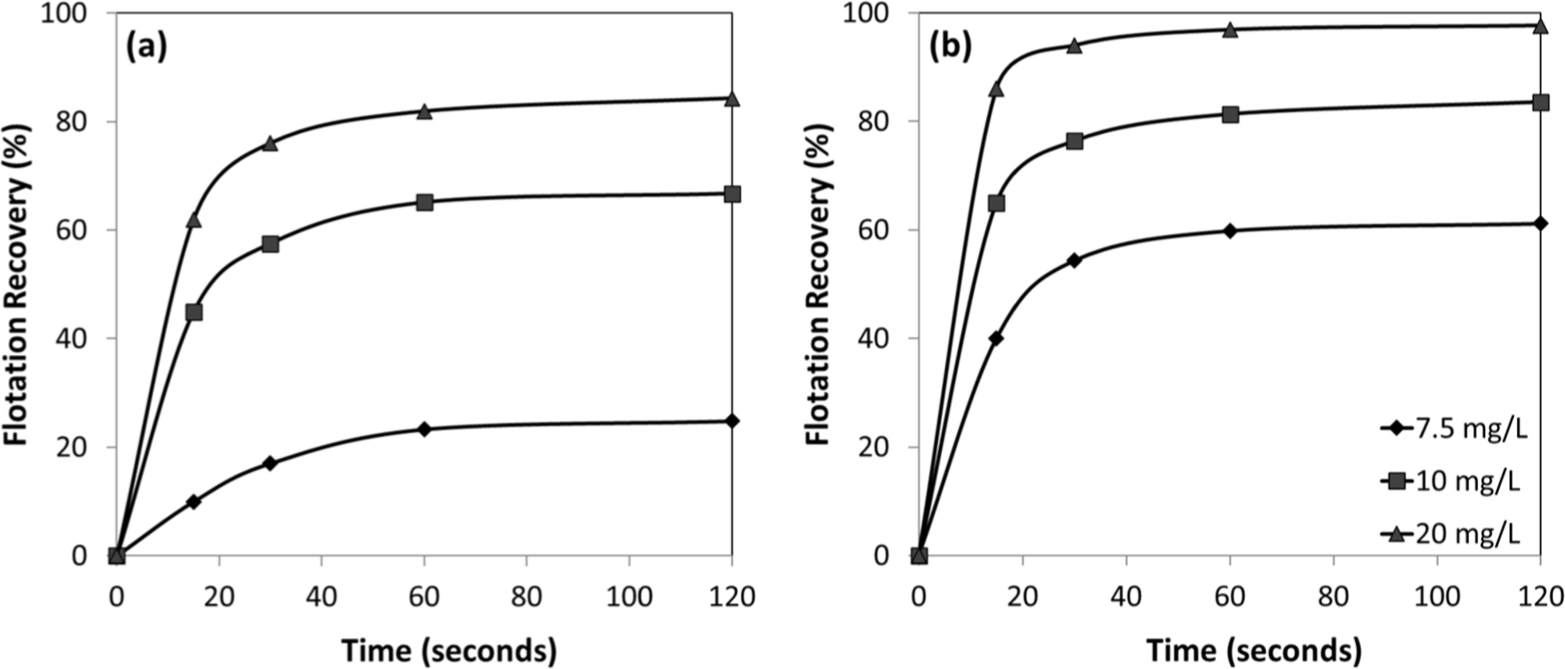
Collector concentration-dependent flotation recovery of (a) chromite and (b) olivine with amine at pH 7–8.

#### 3.2.2 Effect of Sulfonate Type Collector-pH Change on Flotation Efficiency

To investigate an alternative separation strategy, microflotation tests were performed using an anionic sulfonate collector. The pH-dependent behavior of sulfonate exhibited markedly different characteristics compared to the cationic amine system. Analysis of the anionic surfactant sodium dodecyl sulfate (SDS) reveals distinctive speciation behavior across the pH spectrum. Unlike pH-sensitive collectors, SDS predominantly exists in its ionized form (R^–^) throughout the entire pH range, a characteristic attributed to its classification as a strong electrolyte. This property facilitates complete dissociation in aqueous solutions regardless of hydrogen ion concentration, thereby maintaining consistent electrochemical properties across diverse solution conditions.^12,18,19^ This mechanism resulted in high flotation recoveries under strongly acidic conditions; both chromite and olivine demonstrated effective flotation exclusively under strongly acidic conditions (pH 1.5–2.5) when sulfonate was employed (Figure 7a,b). This behavior is consistent with the electrostatic interaction mechanism. Sulfonate exists predominantly in its ionized form (R^–^) across the entire pH range, enabling favorable interactions with mineral surfaces that acquire a positive charge under acidic conditions. Considering that the IEPs of both chromite and olivine are approximately pH 6, their surfaces at pH 1.5–2.5 become strongly positively charged due to protonation of surface hydroxyl groups. This enhanced surface charge strengthens the electrostatic attraction toward the negatively charged sulfonate species, facilitating the formation of stable surface–collector complexes and resulting in improved sulfonate adsorption under strongly acidic conditions. However, flotation recoveries decreased sharply beyond pH 2.5, indicating that effective interaction between sulfonate and the mineral surfaces was confined to very low pH levels, establishing a narrow operational pH range for the sulfonate system.

**Figure 7:**
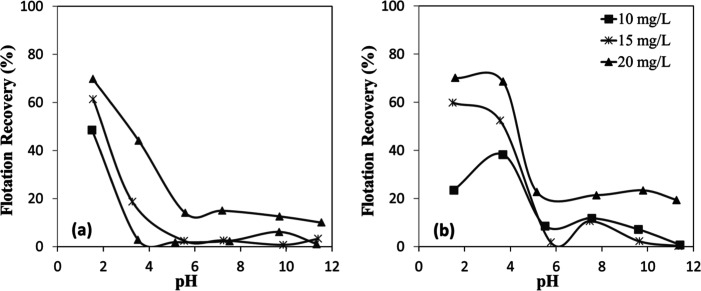
pH-dependent flotation recovery of (a) chromite and (b) olivine with sulfonate.

Despite sharing similar IEP values, olivine’s extended flotation efficiency across a broader pH range could be attributed to its silicate structure. The magnesium and iron ions within olivine’s silicate framework likely formed more stable complexes with sulfonate anions, with Mg^2+^ ions particularly enhancing collector adsorption stability. Conversely, chromite’s spinel structure primarily facilitated electrostatic interactions with limited chemical bonding opportunities, rendering its flotation performance more susceptible to declining surface charge as pH increases beyond 2.5.^20,21^

According to the kinetic tests performed under the given conditions (Figure 6a,b), the flotation responses of chromite and olivine exhibited similar trends, with recoveries increasing as a function of both collector concentration and flotation time. Chromite achieved higher recoveries (Figure 8a) than olivine (Figure 8b) at comparable conditions, particularly at lower collector concentrations. For instance, at 10 mg/L and 30 s, chromite recovery reached ∼41%, whereas olivine showed recovery values less than 20%. At higher collector concentrations, the gap between the two minerals narrowed; at 15 mg/L and 20 mg/L after 120 s, recoveries were about 60% and ∼70% respectively, for both chromite and olivine, indicating nearly comparable flotation efficiency.

**Figure 8:**
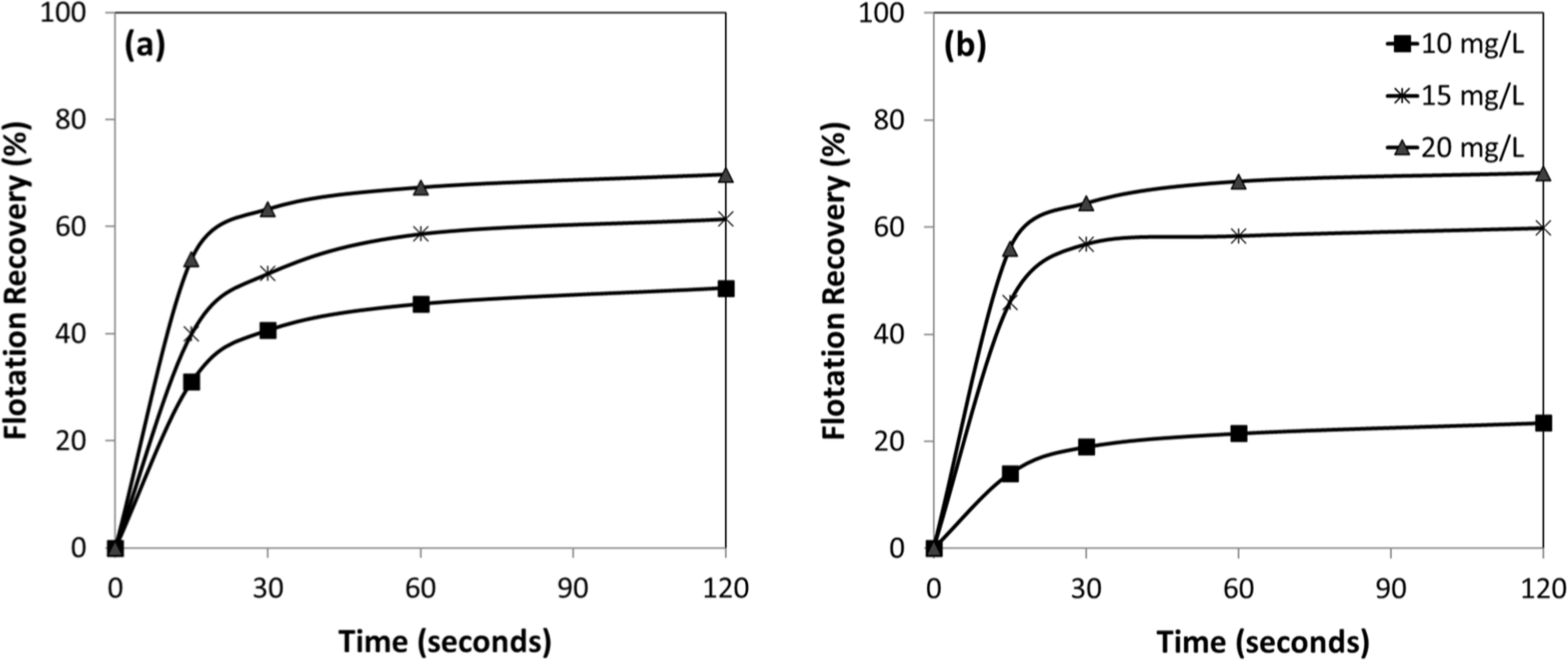
Collector concentration-dependent flotation recovery of (a) chromite and (b) olivine with sulfonate at pH 1.5–2.5.

### 3.3 Effect of Conditioning Time on Flotation Efficiency

The effects of conditioning time on the flotation efficiencies of chromite and olivine were investigated in the presence of the previously determined collector dosage, depending on the variables given in Table 3.

**Table 3:** Variables Applied in Conditioning Time vs pH-Dependent Flotation Tests

**flotation variables**	**values**
mixing rate	1200 rpm (conditioning) 400 rpm (flotation)
AFR	1.6 L/h
flotation periods	15, 30, 60, 120 s
collector concentration	amine 10 mg/L sulfonate 15 mg/L
conditioning time	2.5 min 5 min 10 min

#### 3.3.1 Effect of Conditioning Time-pH Change with Amine on Flotation Efficiency

The influence of pH and conditioning time on the flotation of chromite and olivine using an amine is given in Figure 9a,b. For all conditioning periods, both minerals exhibited a similar flotation response to pH, achieving optimal recoveries at approximately pH 8. At this pH, with optimal conditioning, olivine reached a recovery of ∼83%, while chromite reached ∼65%. However, in the more alkaline region above pH 9, flotation efficiency decreased sharply for both minerals, falling below 45% for olivine and below 30% for chromite. The sharp decline in recovery above pH 9 is consistent with the increasing concentration of hydroxyl ions (OH^–^), which compete with the collector for active sites on the mineral surface, and the potential precipitation of the collector.

**Figure 9:**
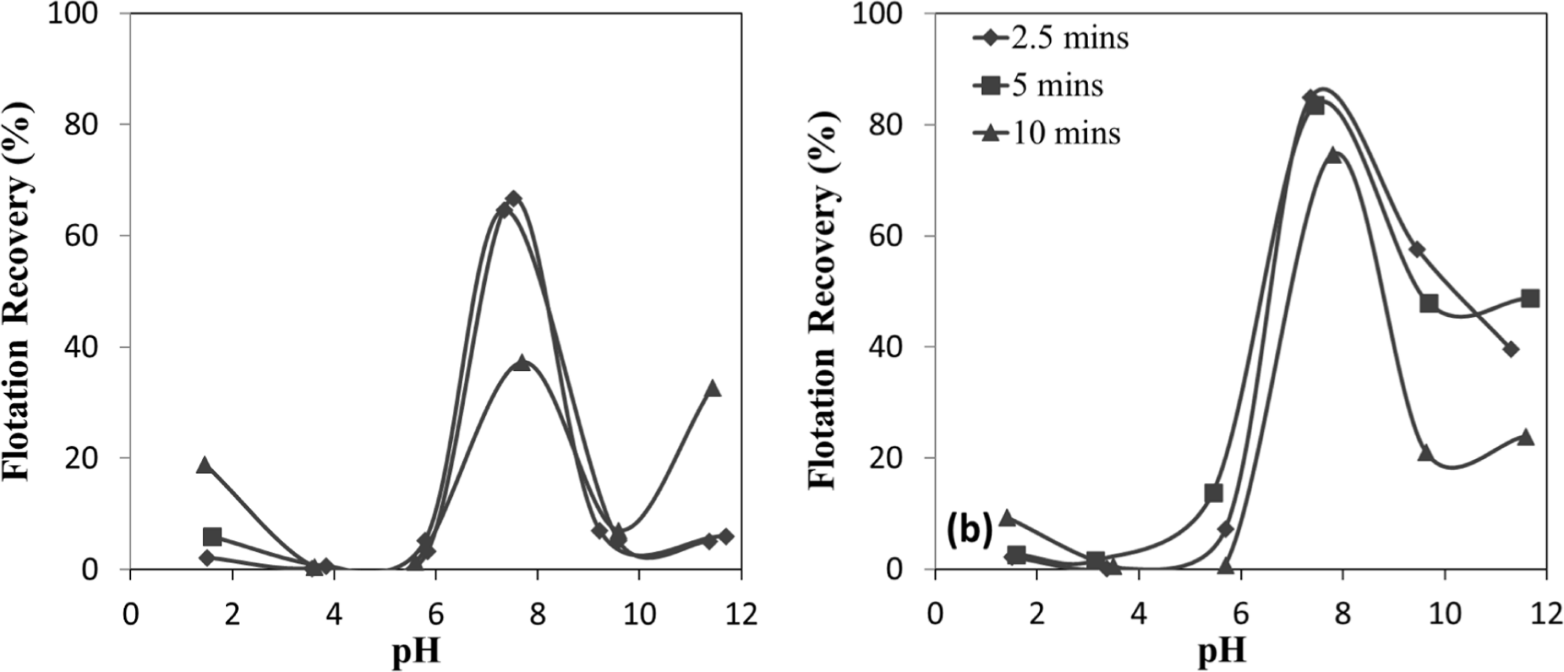
Conditioning time-dependent flotation recovery of (a) chromite and (b) olivine with amine.

Conditioning time exhibited a critical and nonlinear influence on flotation performance. While conditioning periods of 2.5 and 5 min yielded similarly high recoveries, extending the duration to 10 min resulted in a pronounced decline in flotation efficiency. Contrary to the expectation that longer conditioning would enhance mineral–collector interactions, recoveries decreased to below 75% for olivine and below 40% for chromite. This can be attributed to the formation of multilayer adsorption of collectors on the mineral surfaces, leading to partial saturation of active sites and decreased effective collector availability. In this case, excessive conditioning leads to the formation of a second (or more) collector layer adsorbed through tail-to-tail interactions, whereby the hydrophilic polar heads of the outer collector layer orient toward the bulk solution, thereby increasing surface wettability and depressing flotation.^22–25^

Flotation kinetics were also evaluated as a function of conditioning time (Figure 10a,b). The results revealed that the flotation process was remarkably rapid under all tested conditions. For both chromite and olivine, more than 80% of the total floatable fraction was recovered within the first 30 s, indicating that once optimal conditioning was achieved, collector adsorption and subsequent bubble–particle attachment proceeded with high efficiency.

**Figure 10:**
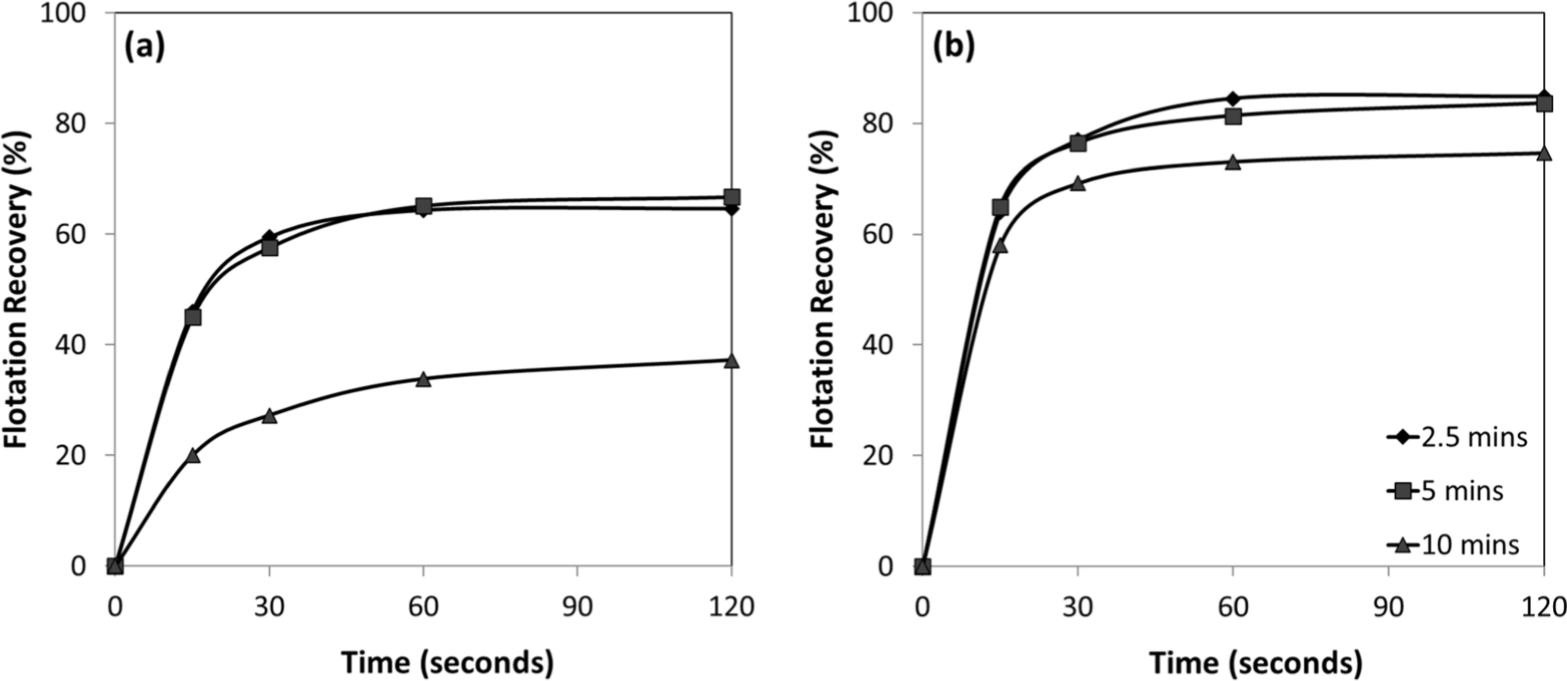
Conditioning time-dependent flotation recovery of (a) chromite and (b) olivine with amine at pH 7–8.

#### 3.3.2 Effect of Conditioning Time-pH Change with Sulfonate on Flotation Efficiency

The flotation responses of chromite and olivine to the anionic sulfonate collector are presented as a function of pH and conditioning time (Figure 11a,b). Both minerals exhibited their highest recoveries under strongly acidic conditions, reaching approximately 65%. The peak recovery for both minerals in a strongly acidic environment is attributed to the strong electrostatic attraction between the anionic sulfonate collector and the positively charged mineral surfaces prevalent at low pH. Conditioning time (2.5, 5, and 10 min) had no discernible effect on the flotation recovery of chromite. In contrast, olivine showed a slight decrease in recovery at a conditioning time of 10 min within the pH range of 3–5.

**Figure 11:**
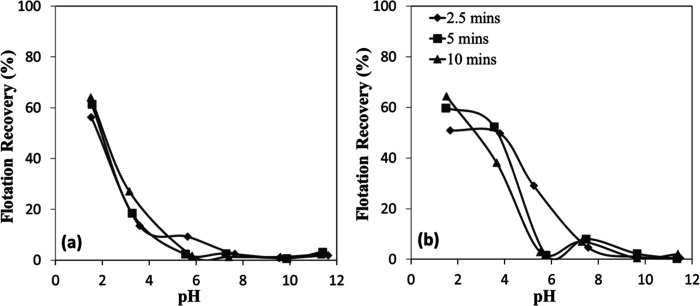
Conditioning time-dependent flotation recovery of (a) chromite and (b) olivine with sulfonate.

In addition, analysis of the flotation kinetics (Figure 12a,b) revealed that the process was extremely rapid and was not significantly influenced by the conditioning time. For both chromite and olivine, approximately 90% of the total floatable material was recovered within the first 30 s. This indicates that collector adsorption was a nearly instantaneous process, rapidly rendering the mineral surfaces hydrophobic and leading to efficient bubble-particle attachment without the need for extended conditioning.

**Figure 12:**
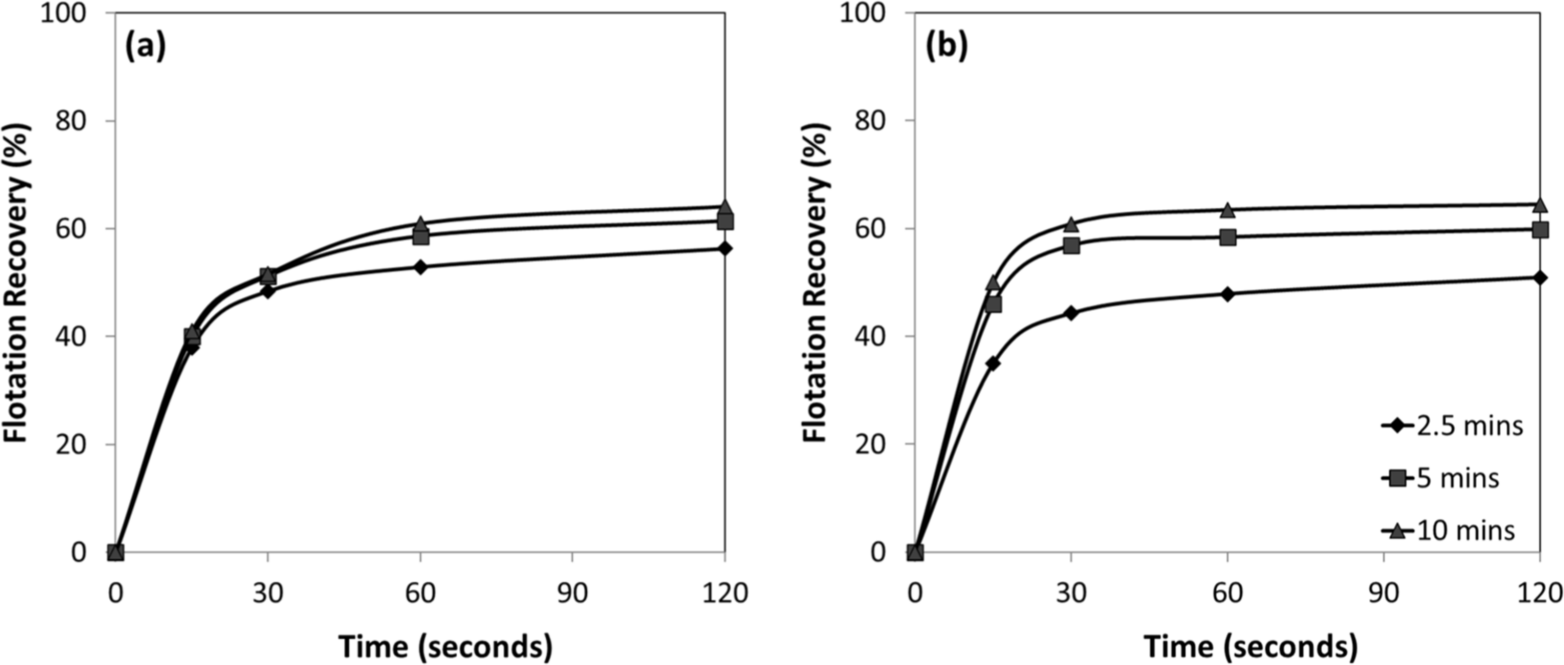
Conditioning time-dependent flotation recovery of (a) chromite and (b) olivine with sulfonate at pH 1.5–2.5.

### 3.4 Effect of AFR on Flotation Efficiency

AFR is a critical operational parameter in flotation, complementing other key variables such as collector concentration, conditioning time, and pulp density. The AFR directly governs the probability of bubble-particle collision and the subsequent transport of hydrophobic minerals to the froth phase. While an increase in aeration can enhance flotation kinetics, an insufficient air supply results in low recovery due to inadequate carrying capacity. Conversely, excessive aeration can induce pulp turbulence, leading to the detachment of particles from bubbles and a subsequent decrease in flotation efficiency. Consequently, identifying an optimal AFR is essential for maximizing recovery and achieving process stability.^26–29^

To investigate this effect and determine the optimal conditions for the system under study, a series of microflotation experiments were conducted. The influence of AFR on the flotation efficiency of chromite and olivine was systematically evaluated. These tests were performed for each collector, using the experimental variables listed in Table 4, allowing for a comprehensive assessment of the interplay between aeration and collector chemistry.

**Table 4:** Variables Applied in AFR vs pH-dependent Flotation Tests

**flotation variables**	**values**
mixing rate	1200 rpm (conditioning) 400 rpm (flotation)
conditioning time	5 min
flotation periods	15, 30, 60, 120 s
collector concentration	amine 10 mg/L sulfonate 15 mg/L
AFR	1.6 L/h 4 L/h 8L/h

#### 3.4.1 Effect of AFR Change with Amine on Flotation Efficiency

The influence of varying AFRs on the flotation performance of chromite and olivine was investigated in the presence of amine (Figure 13a,b). Under constant collector concentration and identical flotation conditions, an increase in AFR enhanced the hydrodynamic environment within the flotation cell. Higher air flow promoted more frequent collisions between mineral particles and air bubbles, resulting in greater hydrodynamic air holdup and, consequently, more efficient particle–bubble attachment. However, the magnitude of this improvement was relatively modest. Even when the AFR was increased 5-fold (from 1.6 L/h to 8 L/h), the corresponding rise in flotation efficiency was only about 10% for both minerals under the same collector concentration. This indicates that flotation performance reaches a plateau beyond a certain AFR, likely due to bubble coalescence or turbulence-induced particle detachment effects.^27–31^

**Figure 13:**
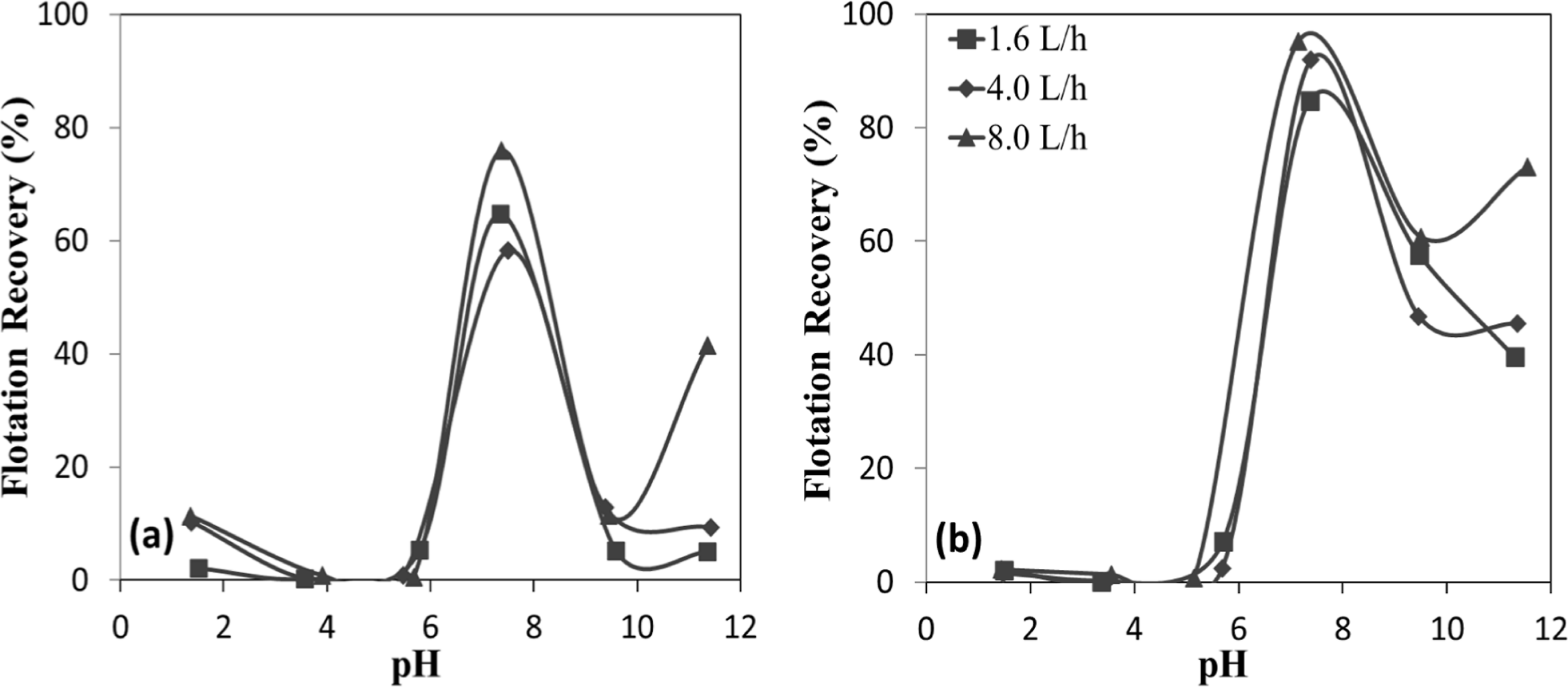
AFR-dependent flotation recovery of (a) chromite and (b) olivine with amine.

Furthermore, the flotation performance of chromite and olivine at near-neutral pH revealed a distinct influence of AFR on mineral recovery. For chromite (Figure 14a), recoveries increased with flotation time but exhibited only marginal improvement beyond 60 s, stabilizing between approximately 64% and 76%, depending on the aeration rate, with the highest values obtained at 8 L/h. In contrast, olivine (Figure 14b) displayed substantially higher recoveries under all conditions, exceeding 85% within the first 30 s and reaching over 94% at an AFR of 8 L/h. Even at the lowest AFR (1.6 L/h), olivine recovery surpassed about 84% after 120 s, indicating a stronger and more rapid flotation response compared to chromite. The results suggest that AFR plays a crucial role in enhancing flotation efficiency, particularly enhancing the separation performance of olivine.

**Figure 14:**
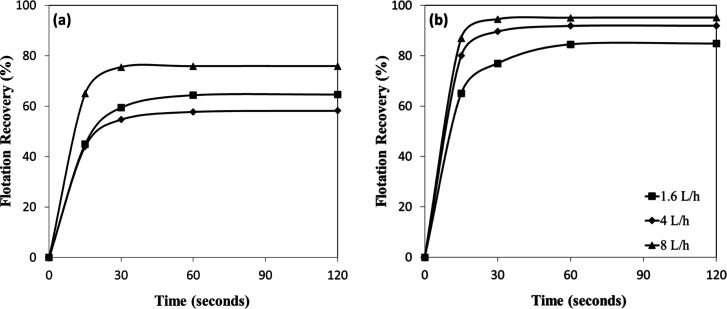
AFR-dependent flotation recovery of (a) chromite and (b) olivine with amine at pH 7–8.

#### 3.4.2 Effect of AFR Change with Sulfonate on Flotation Efficiency

Figure 15a,b shows the influence of different AFRs on the flotation efficiencies of chromite and olivine with sulfonate as a function of pH. The results indicate that changes in AFR had a negligible effect on the flotation efficiency of chromite across the tested range (Figure 15a). In contrast, the flotation efficiency of olivine increased markedly with rising AFR, from approximately 60% at 1.6 L/h to nearly 80% at 8 L/h (Figure 15b). This trend indicates that olivine flotation was more responsive to air flow conditions when using sulfonate as the collector, likely due to improved particle-bubble collision frequency and improved attachment efficiency under higher hydrodynamic air holdup.

**Figure 15:**
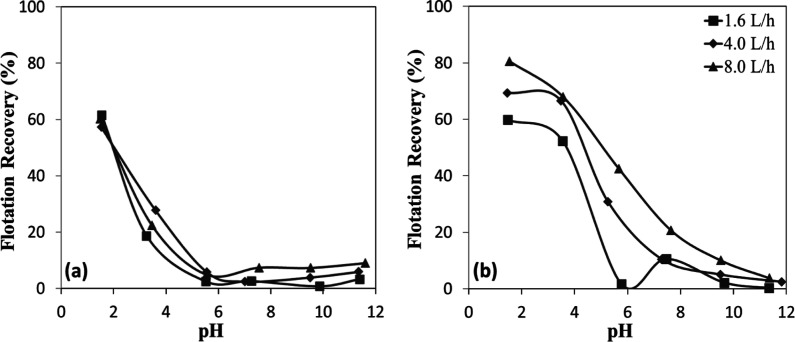
AFR-dependent flotation recovery of (a) chromite and (b) olivine with sulfonate at different pHs.

In Figure 16a,b, the flotation recovery was strongly influenced by the mineral type and AFR. For chromite (Figure 16a), recoveries increased gradually with flotation time but remained relatively modest, reaching approximately 57–61% after 120 s, with only minor variations between aeration rates. In contrast, olivine exhibited consistently higher flotation performance (Figure 16b), achieving about 65% recovery within the first 15 s and exceeding 80% at an AFR of 8 L/h after 120 s. These results indicate that sulfonate monomers interacted more favorably with olivine surfaces, resulting in significantly higher recoveries compared to chromite and thereby providing a potential basis for selective separation. The results also reveal that approximately 90% of the total floated product for both minerals was recovered within the first 30 s of the flotation process, highlighting the rapid kinetics of the process.

**Figure 16:**
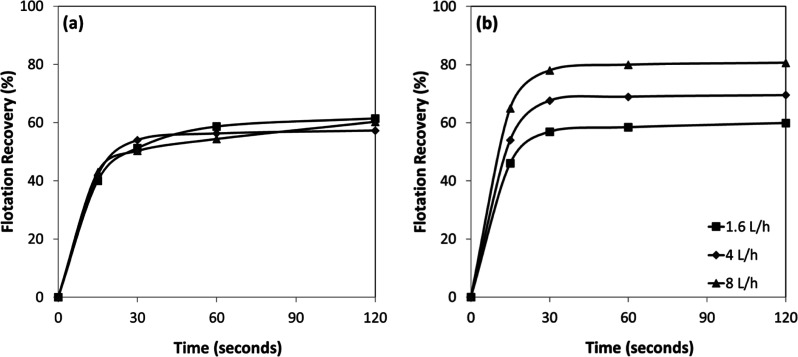
AFR-dependent flotation recovery of (a) chromite and (b) olivine with sulfonate at pH 1.5–2.5.

### 3.5 Effect of Mineral Particle Size on Flotation Efficiency

The relationship between particle size and flotation performance exhibits notable complexity. As mineral particle size decreases, the corresponding increase in surface area enhances reagent adsorption but also elevates reagent consumption, potentially reducing the process’s economic feasibility. Conversely, larger particles are more prone to detachment from air bubbles due to gravitational forces before reaching the froth phase, thereby reducing recovery. This behavior can also be rationalized in terms of surface energy theory. Finer particles possess higher specific surface area and surface energy, which enhances the adsorption of collector molecules and stabilizes particle–bubble attachment. In contrast, coarser particles exhibit lower surface energy and reduced collector adsorption, making them more prone to detachment during flotation. These considerations quantitatively support the observed inverse relationship between particle size and flotation recovery. To systematically investigate this behavior, the flotation responses of chromite and olivine were examined across three narrow particle size fractions, in conjunction with the other flotation parameters summarized in Table 5. This size-dependent investigation provided crucial insights for optimizing the chromite–olivine separation process, addressing a fundamental challenge in mineral processing: determining the optimal particle size range that maximizes both selectivity and recovery while minimizing reagent consumption.

**Table 5:** Variables Applied in Particle Size vs pH-Dependent Flotation Tests

**flotation variables**	**values**
mixing rate	1200 rpm (conditioning) 400 rpm (flotation)
conditioning time	5 min
AFR	1.6 L/h
flotation periods	15, 30, 60, 120 s
collector concentration	amine 10 mg/L sulfonate 15 mg/L
particle size	–300 + 212 μm −212 + 150 μm −150 + 106 μm

#### 3.5.1 Effect of Mineral Particle Size Change with Amine on Flotation Efficiency

The relationship between mineral particle size and flotation efficiency was investigated for chromite and olivine minerals using an amine as a function of pH (Figure 17a,b). The results revealed that flotation efficiency was strongly dependent on particle size, even under identical flotation conditions for both minerals.^32^ For both chromite and olivine, flotation recovery exhibited an inverse relationship with particle size, reaching approximately 90% for the finest fractions. In contrast, the coarsest particle size fraction (−300 + 212 μm) displayed markedly poor flotation performance (<10%). This behavior can be attributed to the mechanical limitations of the flotation process, wherein larger particles, although initially attached to air bubbles and transported toward the froth phase, tend to detach due to gravitational forces and turbulent flow before reaching the froth layer. This observation is consistent with classical flotation theory, which suggests an optimal size range for efficient mineral recovery.

**Figure 17:**
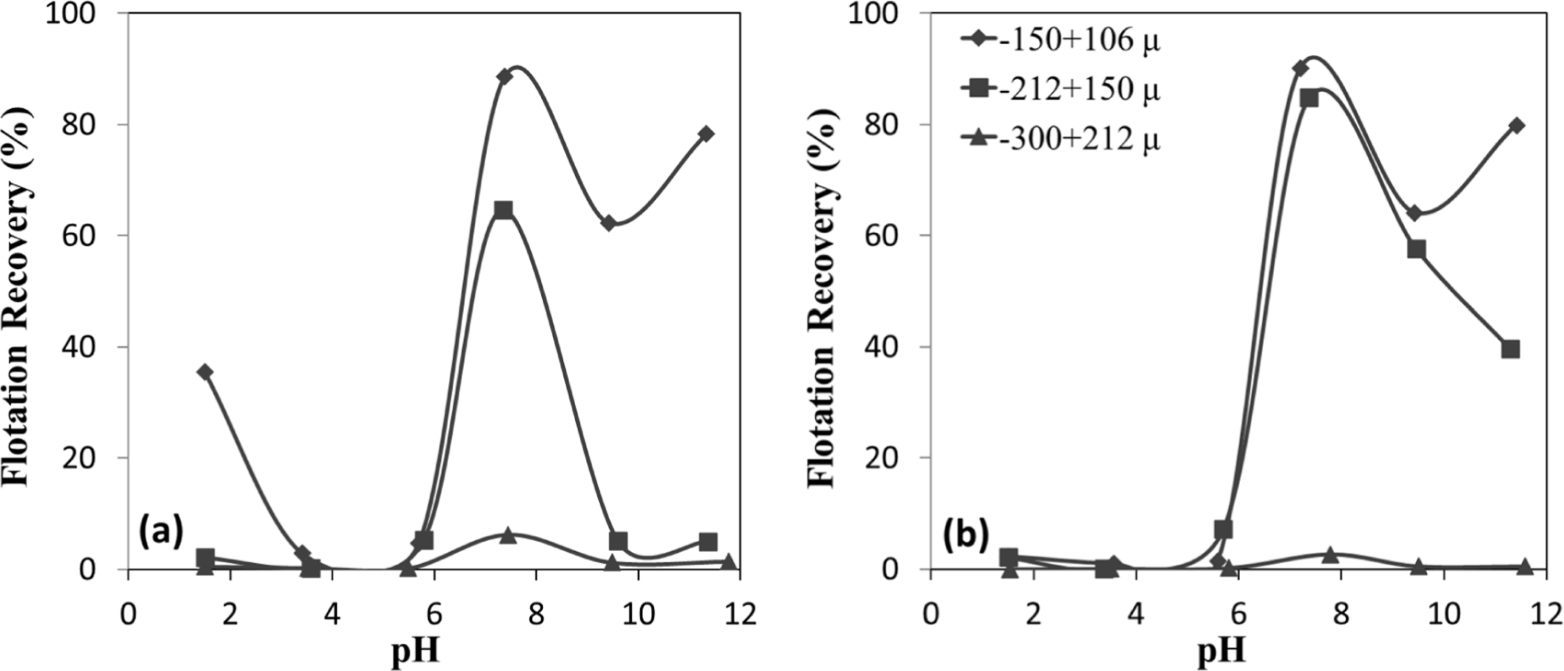
Particle size-dependent flotation recovery of (a) chromite and (b) olivine with amine at different pHs.

Temporal analysis of the flotation process (Figure 18a,b) revealed that more than 85% of the total concentrate for both minerals was recovered within the initial 30 s of flotation time, indicating rapid kinetics of the collector–mineral interaction. Interestingly, while chromite exhibited a consistent increase in flotation efficiency with decreasing particle size (Figure 18a), olivine showed similar flotation efficiencies for the intermediate size fractions (−212 + 150 μm and −150 + 106 μm) (Figure 18b). This difference in behavior suggests mineral-specific interactions with the amine collector, influenced by variations in surface chemistry, mineral density, or particle morphology.

**Figure 18:**
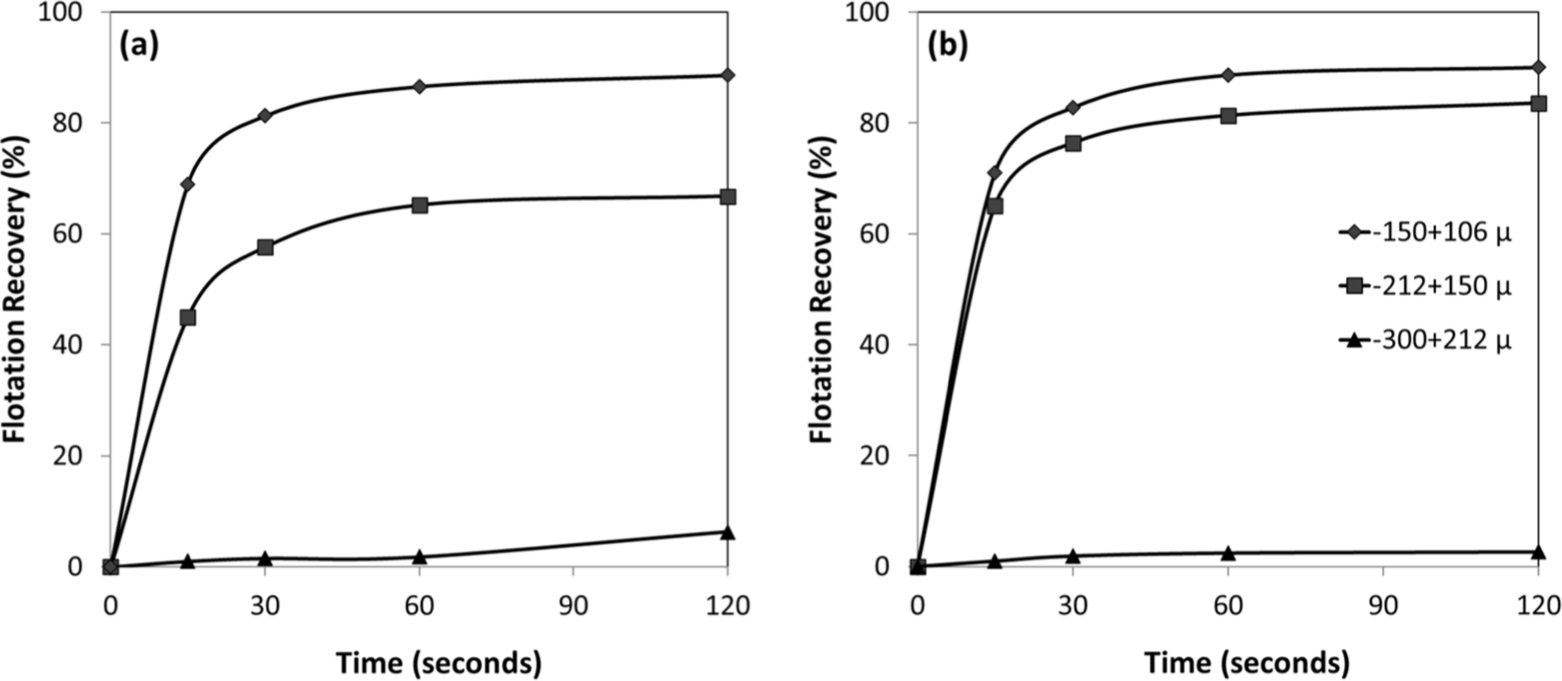
Particle size-dependent flotation recovery of (a) chromite and (b) olivine with amine at pH 7–8.

#### 3.5.2 Effect of Mineral Particle Size Change with Sulfonate on Flotation Efficiency

The microflotation experiments conducted with shorter C–H chain sulfonate-type collectors revealed significant insights into the flotation behavior of chromite and olivine minerals (Figure 19a,b). Analysis of the data demonstrates a consistent trend where flotation efficiency increased with decreasing particle size for both minerals. This inverse relationship between particle size and recovery efficiency reinforces the fundamental principle that finer particles generally exhibit enhanced flotation performance due to improved bubble-particle attachment dynamics. The rapid decline in flotation efficiencies with increasing mineral particle size could be attributed to insufficient adhesion forces between the sulfonate-treated mineral surfaces and air bubbles. Larger mineral particles, despite successful initial attachment to bubbles, appeared unable to maintain this attachment against gravitational forces during ascent through the pulp phase. This detachment mechanism became increasingly pronounced with larger particle sizes, resulting in substantially reduced recovery for coarser fractions of both chromite and olivine.

**Figure 19:**
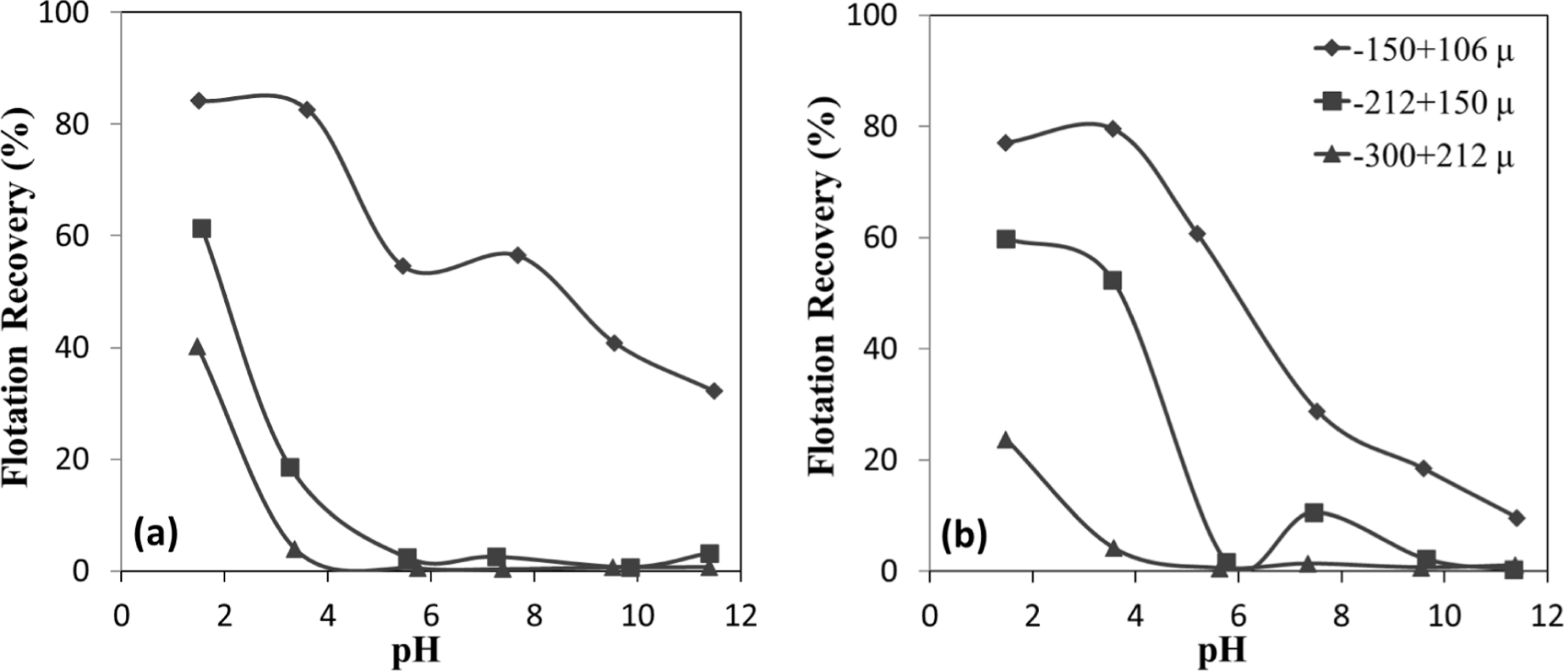
Particle size-dependent flotation recovery of (a) chromite and (b) olivine with sulfonate at different pHs.

Furthermore, finer particle size fractions exhibited improved recoveries across nearly the entire pH range, which can be attributed to their increased surface area and enhanced collector adsorption. Notably, both chromite and olivine achieved effective flotation even under moderately acidic to near-neutral conditions. Below the IEP (pH < 6), surface hydroxyl groups are protonated (M–OH_2_^+^), generating positive charges that strongly attract sulfonate ions, resulting in sharply increased recoveries under acidic conditions,^33^ especially in the finest fraction (−150 + 106 μm), where chromite reached ∼84% (Figure 19a) and olivine ∼77% (Figure 19b) at pH ∼1.5. Importantly, particle size reduction not only increased the effective surface area but also created unsaturated adsorption sites during grinding, providing additional active centers for collector attachment. Olivine’s higher flotation recoveries than chromite at mildly acidic pH (3–4) with −212 + 150 μm-sized samples were likely due to differences in surface site density and Mg^2+^/Fe^2+^ dissolution behavior favoring sulfonate adsorption. Overall, the enhanced flotation of finer particles across nearly the entire pH range can be explained by a combination of increased surface area, higher surface energy, and the creation of unsaturated adsorption sites during grinding, which collectively improve collector adsorption and bubble attachment stability. These mechanistic insights provide experimental evidence supporting the observed particle size effects, explaining the decreased recovery of coarser fractions and the relatively high flotation recoveries of fine particles (40–60%) under near-neutral to weakly alkaline conditions, in contrast to the negligible flotation of coarser fractions.

The time-dependent flotation recoveries reveal that both chromite and olivine exhibited markedly improved recoveries with decreasing particle size (Figure 20a,b). For chromite (Figure 20a), recoveries after 120 s increased from ∼40% in the coarse fraction (−300 + 212 μm) to about 60% in the intermediate fraction (−212 + 150 μm) and about 84% in the finest fraction (−150 + 106 μm). A similar trend was observed for olivine (Figure 20b), with final recoveries of about ∼25%, ∼60%, and ∼77% for the same size fractions, respectively. The kinetics also indicate that finer particles reached higher flotation efficiencies more rapidly, as reflected by the steep increase within the first 30 s.

**Figure 20:**
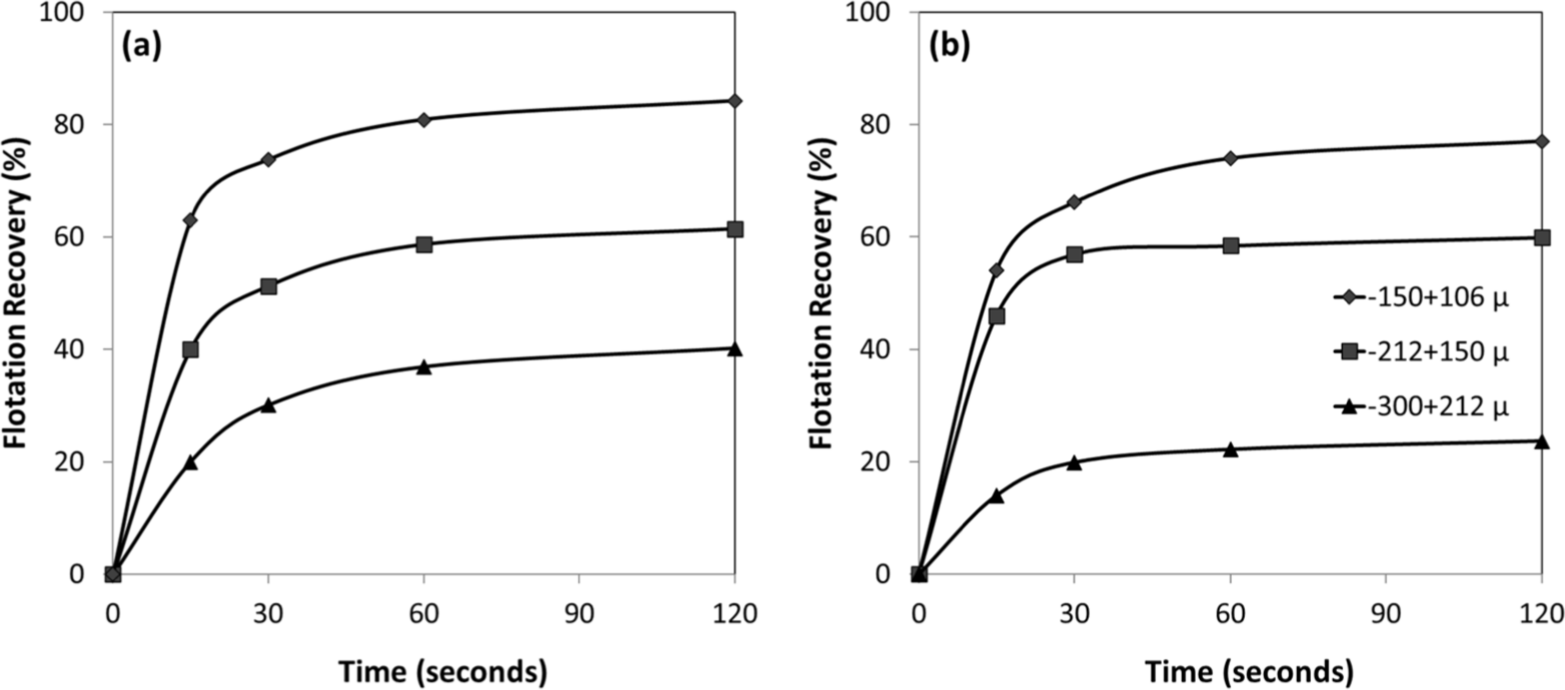
Particle size-dependent flotation recovery of (a) chromite and (b) olivine with sulfonate at pH 1.5–2.5.

### 3.6 TGA Analysis

To assess the interactions between the collectors and mineral surfaces and to facilitate a comparative evaluation of their thermal behavior, TGA analyses were conducted on both untreated collector solutions and those conditioned separately with chromite and olivine (sized in −212 + 150 μm), at their respective optimum pH values (pH 7–8 for amine and pH 1.5–2.5 for sulfonate). The analyses were performed under the key experimental conditions defined in Table 5. To clearly identify the thermal effects resulting from the conditioning process and possible interactions between the collector molecules and mineral surfaces, the obtained TGA profiles were compared with those of distilled water used in the preparation of the dilute collector solutions under identical experimental conditions (Figures 21 and 22). This comparison enabled the evaluation of variations in thermal stability and decomposition characteristics resulting from mineral–collector interactions under optimal conditioning conditions.

**Figure 21:**
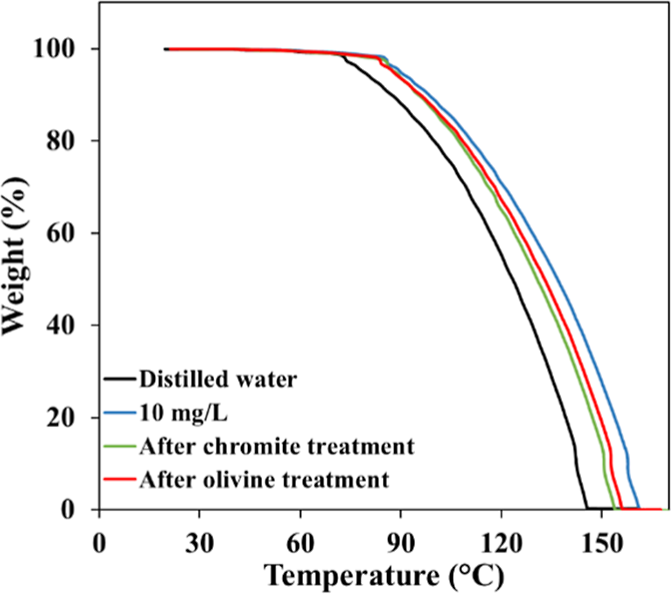
TGA profile of amine solution before/after mineral treatment at pH 7–8.

**Figure 22:**
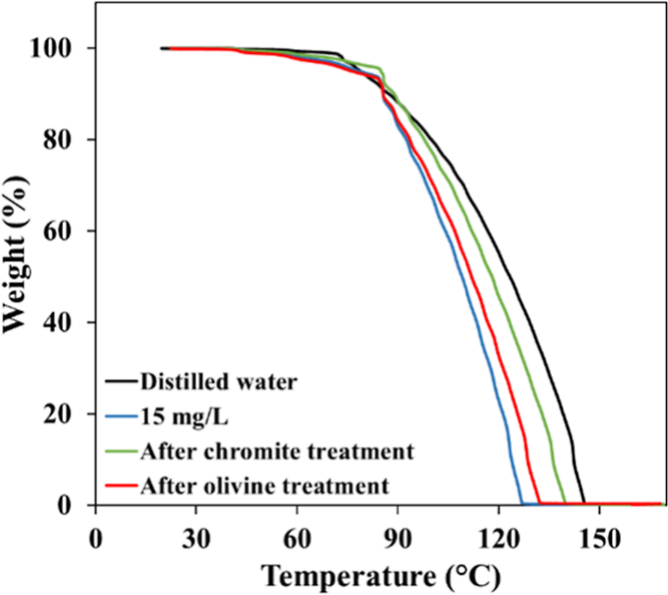
TGA profile sulfonate solution before/after mineral treatment at pH 1.5–2.5.

TGA profile of distilled water reveals that the onset of significant mass loss occurs at approximately 70 °C, progressively accelerating with increasing temperature, until the sample is fully converted to the gaseous phase near 145 °C. This thermal behavior corresponds to the characteristic evaporation process of distilled water, and the obtained results show good agreement with previously reported literature data.^34^

TGA of the diluted amine solution (10 mg/L in distilled water, at pH 7–8) indicated that mass loss initiated at approximately 85 °C and was nearly completed by around 160 °C (Figure 21). This behavior is predominantly attributed to the evaporation of water and the minor fraction of volatile amine species, rather than to thermal degradation. When the amine collector solution was conditioned with the mineral samples, a distinct shift in the mass-loss trend was observed. For the chromite-conditioned sample, gradual mass loss occurred between 45 and 85 °C, reaching approximately 97% of the total mass, followed by a rapid decline that completed around 155 °C. After olivine-treatment, the TGA curve exhibited a similar trend to that of chromite up to 85 °C; however, beyond this point, a less pronounced decrease was recorded, with the mass-loss process completing near 157 °C. These results reveal that, following conditioning with the minerals, the reduction in free amine monomers in solution—due to their interaction and adsorption onto the mineral surfaces—led to a mass-loss behavior that increasingly resembled that of distilled water.

In contrast to the amine solution, TGA analyses conducted with sulfonate (15 mg/L) exhibited a distinctly different thermal behavior (Figure 22). Relative to distilled water, the dilute sulfonate solution showed an earlier onset of mass loss, initiating at approximately 45 °C. The process progressed rapidly, reaching about 92% of the total mass loss by 85 °C and completing near 128 °C. This pattern reflects the higher volatility and thermal sensitivity of sulfonate species in aqueous media. Upon conditioning with the minerals, a clear shift in the TGA profiles was observed. The interaction and adsorption of sulfonate monomers onto the mineral surfaces reduced the concentration of free collector in the supernatant, resulting in a mass-loss behavior that increasingly resembled that of distilled water. Consequently, the completion of the mass-loss process occurred at higher temperatures—around 141 °C following chromite treatment and approximately 133 °C after olivine treatment.

### 3.7 FTIR Analysis

FTIR-ATR analyses were conducted to investigate the interactions between chromite, olivine, and the collectors employed in this study, with particular emphasis on the pH conditions that yielded the highest flotation performance. These measurements aimed to clarify both the nature and the strength of the interactions between the collectors and the mineral surfaces. Accordingly, spectra were acquired in the mid-infrared region (4000–400 cm^–1^) for pure collectors, collector-treated mineral particles, and untreated mineral particles.

#### 3.7.1 FTIR Analysis with Amine-Type Collector

In Figure 23a,b, the long-chain amine exhibited characteristic IR absorption bands in the 3000–2800 cm^–1^ region, which can be assigned to the stretching vibrations of C–H bonds in the alkyl chain. Specifically, the absorption at 2957 cm^–1^ corresponds to the asymmetric stretching of terminal −CH_3_ groups, while the band at 2922 cm^–1^ is attributed to the asymmetric stretching of –CH_2_– groups. The peak observed at 2853 cm^–1^ arises from the symmetric stretching of −CH_2_– units. In addition, several weaker absorption features were detected within the fingerprint region, corresponding primarily to N–H stretching vibrations of the amine functional group and O–H vibrations, which may be associated with adsorbed moisture or hydrogen bonding effects.^35,36^ On the other hand, the FTIR spectrum of chromite (Figure 23a) is mainly characterized by metal–oxygen vibrations within the spinel structure. Distinct absorption bands are typically observed as Cr–O and Fe–O stretching vibrations between 650 and 400 cm^–1^ and as Cr=O between 1000 and 800 cm^–1^.^37–39^ In contrast, the FTIR spectrum of olivine (Figure 23b) is dominated by silicate vibrations. Strong absorption bands occur in the range of 1100–900 cm^–1^ due to asymmetric Si–O stretching, while symmetric Si–O stretching modes are observed near 850–800 cm^–1^. Additional Si–O bending vibrations appear in the 600–400 cm^–1^ region.^40–42^ Furthermore, weak O–H stretching bands may be present in the 3700–3400 cm^–1^ region, which are generally associated with structural hydroxyl groups or adsorbed moisture.

**Figure 23:**
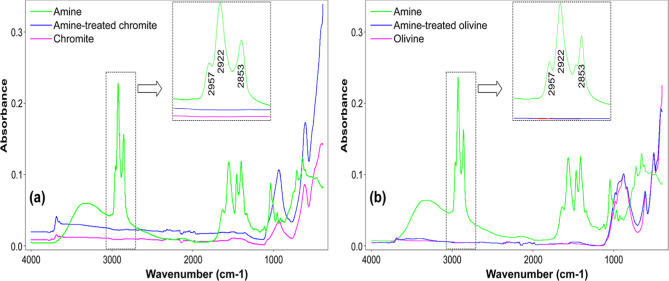
FTIR spectra of (a) chromite and (b) olivine before/after treatment with amine.

Considering the pH range of 7–8, where the highest flotation efficiencies of chromite and olivine were achieved with the use of cationic amine-type collectors, FTIR analyses were conducted to facilitate comparison and to elucidate the interactions between the collector and mineral samples. For this purpose, spectra of amine-treated mineral particles, the pure amine collector, and untreated mineral particles were obtained separately at the optimum collector concentration determined within the pH range of 7 and 8, and the results are presented together in Figure 23a,b. The findings indicated that an electrostatic interaction occurred between the negatively charged chromite and olivine surfaces and the positively charged collector monomers (RNH_3_^+^), which is consistent with the zeta potential measurements and the determined maximum flotation efficiencies. However, these weak electrostatic bonds were removed upon washing the amine-treated mineral particles with distilled water. Furthermore, in agreement with previous literature,^43^ no additional FTIR peak, asymmetric and symmetric stretching vibrations of the CH_2_ group, corresponding to collector–mineral interactions, was determined within the range of 2957–2853 cm^–1^ (highlighted as the shaded region in each figure).

#### 3.7.2 FTIR Analysis with Sulfonate-Type Collector

The FTIR spectrum of petroleum sulfonate exhibited characteristic absorption bands corresponding to the sulfonate functional group and the hydrocarbon chains (Figure 24a,b). Asymmetric and symmetric S=O stretching vibrations were observed at 1040–1080 cm^–1^ and 1170–1190 cm^–1^, respectively, while C–H bending and stretching vibrations of −CH_3_ and −CH_2_ groups appeared at 1375–1385, 1460–1470, and 2850–2925 cm^–1^. A weak broad band around 3400 cm^–1^ was attributed to O–H stretching from moisture. These spectral features confirm the molecular structure of petroleum sulfonate and are consistent with the previous reports.^44–46^ Considering the zeta potential values and the pH range (1.5–2.5) corresponding to the maximum flotation efficiencies of chromite and olivine, the petroleum sulfonate remained ionic across all tested pH values and exhibited electrostatic interactions with the positively charged mineral surfaces within this range. However, these weak interactions were removed upon washing the collector-treated mineral particles with distilled water. As indicated in the shaded regions of the spectra, no additional C–H peaks corresponding to asymmetric and symmetric stretching vibrations of the CH_2_ group were observed, confirming the absence of strong interactions between the sulfonate and the mineral particles (Figure 24a,b).

**Figure 24:**
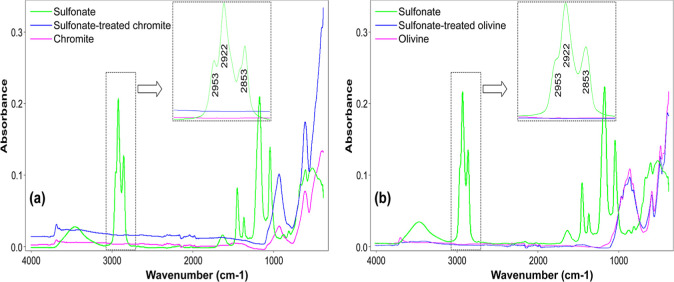
FTIR spectra of (a) chromite and (b) olivine before/after treatment with sulfonate.

## 4 Conclusion

This study presents a comprehensive comparative evaluation of the flotation behavior of chromite and olivine, two minerals that pose significant separation challenges due to their similar surface properties and fine liberation under conventional beneficiation practices. Systematic microflotation experiments, complemented by TGA and FTIR analyses, were conducted to thoroughly examine the influence of collector chemistry, concentration, pulp pH, AFR, conditioning time, and particle size on flotation performance.

The results demonstrated that both collector chemistry and operational parameters significantly influence flotation efficiency and selectivity. Amine exhibited comparable flotation behavior for chromite and olivine, achieving maximum recoveries of approximately 80% and 90%, respectively, at neutral pH, although olivine displayed higher recoveries at elevated pH and lower collector concentrations. In contrast, sulfonate proved highly effective in promoting rapid and selective attachment to mineral surfaces under acidic conditions, with optimal performance observed at pH < 2, yielding recoveries exceeding 83% for chromite and 94% for olivine. Flotation kinetics were consistently fast in all cases, with the majority of recoverable particles collected within the first 30 s.

Flotation efficiencies were further improved by increasing the AFR, while particle size reduction played a critical role in accelerating flotation kinetics and enhancing ultimate recovery for both chromite and olivine. In contrast, coarser particle sizes resulted in a marked decline in recovery, dropping below 10% under certain conditions. Notably, the sulfonate collector exhibited a broader pH range of high flotation efficiency with decreasing particle size—a trend not observed for amine—highlighting the stronger dependence of sulfonate adsorption on both pH-controlled surface charge and the availability of unsaturated adsorption sites generated by grinding.

TGA analyses demonstrated that the adsorption of amine and sulfonate collectors onto chromite and olivine surfaces markedly modifies their thermal behavior, reducing the concentration of free monomers and shifting mass-loss profiles toward those of distilled water. These findings confirm that TGA effectively captures mineral–collector interactions and highlight the distinct adsorption characteristics of the two collectors under their respective optimal pH conditions.

FTIR analyses showed no distinct collector-derived C–H absorption peaks on minerals conditioned with collectors and subsequently rinsed, indicating that adsorption is predominantly physical rather than chemical. This suggests that the observed flotation selectivity arises mainly from surface charge modification and hydrophobic interactions, rather than from strong chemical bonding.

Overall, this study enhances the mechanistic understanding of chromite–olivine flotation and delineates the operational conditions that facilitate their selective separation. The results offer a solid basis for optimizing collector type, concentration, and pH in both chromite processing plants and tailings flotation, with the potential to improve selectivity while reducing reagent consumption. Furthermore, the findings provide practical guidance for laboratory-scale optimization and strategies for recovering chromite and olivine from fine-grained tailings, thereby contributing to more efficient resource utilization and promoting sustainable mineral beneficiation practices.
